# Conversion Therapy Exposure and Elevated Cardiovascular Disease Risk

**DOI:** 10.1001/jamanetworkopen.2025.8745

**Published:** 2025-05-06

**Authors:** James K. Gibb, Joshua M. Schrock, Madison Shea Smith, Richard T. D’Aquila, Thomas W. McDade, Brian Mustanski

**Affiliations:** 1 Department of Anthropology, Northwestern University, Evanston, Illinois; 2 Institute for Policy Research, Northwestern University, Evanston, Illinois; 3 Institute for Sexual and Gender Minority Health and Wellbeing, Northwestern University, Chicago, Illinois; 4 Department of Medical Social Sciences, Northwestern University, Chicago, Illinois; 5 Division of Infectious Diseases, Department of Medicine, Feinberg School of Medicine, Northwestern University, Chicago, Illinois

## Abstract

**Importance:**

Sexual orientation and gender identity change efforts (SOGICE), commonly known as conversion therapy, persist across the United States despite having been discredited due to their harmful mental health impact. Understanding the health consequences of SOGICE is critical, particularly for conditions exacerbated by stress, such as cardiovascular disease.

**Objective:**

To investigate the association between SOGICE exposure and cardiovascular disease risk.

**Design, Setting, and Participants:**

This cohort study draws on data from the observational cohort study RADAR, which included sexual and gender minority (SGM) young adults assigned male at birth (AMAB) in Chicago, Illinois. All data for this analysis were collected cross-sectionally during the same study visit for each participant between December 1, 2023, and October 31, 2024. Participants who completed SOGICE exposure measures and had cardiovascular health data were eligible; participants with incomplete data were excluded.

**Exposure:**

SOGICE exposure and duration.

**Main Outcomes and Measures:**

Primary outcomes were diastolic blood pressure (DBP) and systolic blood pressure (SBP) measured via automatic monitors, systemic inflammation (SI) assessed through plasma biomarkers, and self-reported hypertension or high blood pressure (HHBP) diagnosis. Regression analyses were adjusted for sociodemographic and behavioral covariates.

**Results:**

Among 703 participants (mean [SD] age, 26.75 [4.60] years), 540 (76.8%) identified as cisgender, and 163 (23.2%) identified as transgender or gender diverse. Seventy-two participants (10.2%) reported exposure to SOGICE. Exposed participants had higher SI levels (β = 0.83; 95% CI, 0.17-1.49; *P* = .02), increased DBP (β = 3.46; 95% CI, 0.60-6.32; *P* = .02), elevated SBP (β = 4.28; 95% CI, 0.68-7.87; *P* = .02), and greater odds of self-reported HHBP diagnosis (odds ratio, 2.86; 95% CI, 2.19-3.54; *P* = .003) compared with nonexposed participants. Associations remained significant after adjusting for covariates.

**Conclusions and Relevance:**

In this cohort study of SGM young adults AMAB, exposure to SOGICE was associated with adverse cardiovascular health indicators, including elevated DBP and SBP, increased SI, and higher odds of self-reported HHBP. These findings underscore the need for public health and policy interventions to enforce and strengthen bans on SOGICE practices. Recognizing the cardiovascular health consequences of SOGICE contributes to addressing SGM health disparities and emphasizes the importance of affirmative care approaches that prioritize the well-being of SGM individuals.

## Key Points

**Question:**

Are exposures to sexual orientation and gender identity change efforts (SOGICE) associated with increased risk of cardiovascular disease?

**Findings:**

In this cohort study of 703 sexual and gender minority young adults assigned male at birth, SOGICE exposure was associated with elevated blood pressure, increased systemic inflammation, and higher odds of self-reported hypertension diagnosis.

**Meaning:**

These results suggest that exposure to SOGICE may increase the risk of adverse cardiovascular health outcomes, underscoring the need for enforcement of bans and affirmative care approaches.

## Teaser

This cohort study assesses whether exposure to sexual orientation and gender identity change efforts is associated with cardiovascular disease risk in sexual and gender minority young adults assigned male at birth.

## Introduction

Sexual orientation and gender identity change efforts (SOGICE), also known as conversion or reparative therapy, encompass practices used to change same-sex attractions and behaviors and/or sexual minority (eg, lesbian, gay, and bisexual) and gender minority (eg, transgender and nonbinary) identities.^1,2^ These interventions, rooted in discredited beliefs that sexual and gender minority (SGM) identities, behaviors, and/or attractions are immoral, abnormal, and/or pathological, have been unequivocally denounced by leading medical, psychological, and human rights organizations based on their harmful psychological impacts.^1,3,4,5,6^ The detrimental psychological effects of SOGICE include heightened risk of depression, anxiety, and suicidality among SGM individuals.^7,8,9,10,11,12,13,14,15,16,17^ Although most of the research to date has examined the effects of sexual orientation change efforts (SOCE) on the mental health of sexual minority groups, there is an emerging literature investigating the harmful effects of gender identity change efforts on the health of gender minority groups.^10^

Despite efforts toward eradication, SOGICE continues to pose substantial ethical and health-related challenges (eFigure 1 in Supplement 1). Currently, 23 states and the District of Columbia have laws banning SOGICE, 5 states have laws partially banning the practice, and 18 states have no laws banning SOGICE.^18^ Furthermore, Indiana has a law prohibiting local-level bans of SOGICE, and 3 states (Alabama, Florida, and Georgia) have court rulings outlawing bans on SOGICE therapy.^18^ More than 1320 practitioners of SOGICE are currently operating in the US; 46% hold active professional licenses, and 54% operate within religious institutions.^19^ Notably, many of these practitioners operate in states where SOGICE is banned. For example, despite a state-level ban in Illinois, a recent survey by The Trevor Project^19^ identified more than 50 SOGICE practitioners in the state.

Increasingly, research has shown that SOGICE exposure is associated with adverse mental health outcomes among SGM individuals, including increased risks of depression, anxiety, and suicidality.^13,15^ However, investigation into the long-term physical health consequences of SOGICE exposure, particularly concerning cardiovascular health and systemic inflammation (SI), remains scant. Meyer minority stress theory posits that SGM individuals experience chronic stress due to social stigma, prejudice, and discrimination, which in turn contributes to adverse health outcomes.^20^ Within this framework, SOGICE functions as a particularly insidious form of minority stress, reinforcing both internalized and experienced stigma and thereby causing additional chronic stress among SGM individuals.^21,22,23^ Chronic stress is known to activate the hypothalamic-pituitary-adrenal axis and the sympathetic nervous system, triggering biological responses that elevate blood pressure and SI—both principal contributors to cardiovascular disease risk.^24,25,26^ Consequently, SGM populations are at elevated risk for cardiovascular disease^27,28,29,30,31,32^ as well as SI.^33,34,35^ Moreover, SGM individuals are especially likely to experience SOGICE during later childhood, adolescence, and young adulthood—critical periods of growth and development.^15,36,37,38^ Exposure during these stages may be particularly harmful, increasing the risk of developing adverse health outcomes later in adulthood.^39,40,41^ Our study aims to investigate the association between SOGICE exposure and blood pressure, SI, and self-reported diagnosis of hypertension or high blood pressure (HHBP), important measures of cardiovascular disease risk, in a diverse cohort of young SGM adults assigned male at birth (AMAB).

## Methods

### Setting, Design, and Study Populations

The data for this study were collected as part of RADAR, an observational cohort study of young men who have sex with men, transgender women, and nonbinary individuals.^42^ Participants aged 16 to 29 years are evaluated every 6 months for HIV risks, substance use, relationship and sexual partner characteristics, and social and psychological measures. Although RADAR is a longitudinal cohort study, all data for this analysis were collected cross-sectionally during the same study visit for each participant between December 1, 2023, and October 31, 2024. Beginning in late 2023, RADAR participants were asked about their past experiences with SOGICE. Cardiovascular health measures and covariates were collected at the same study visit for each participant or, if unavailable, data from the next most recent study visit were used. The initial dataset included 743 participants. All participants provided written consent at each study visit. This study adheres to the Strengthening the Reporting of Observational Studies in Epidemiology (STROBE) reporting guideline for epidemiologic studies to ensure transparency and methodologic rigor.^43^ All study protocols were approved by the Northwestern University institutional review board.

To ensure data quality and reduce the influence of extreme values, we applied the Tukey method to identify and remove outliers for blood pressure and SI measures. Outliers were defined as values outside 1.5 times the IQR and were excluded separately for each outcome variable in line with a pairwise deletion approach. This process resulted in the removal of 17 participants with extreme SI values and 12 participants with extreme blood pressure values, enhancing statistical validity by reducing potential skewness and variance inflation. After the outlier exclusions, we performed a listwise deletion of cases (n = 11) with missing data for our primary variable of interest (SOGICE exposure). This process yielded a final analytic sample of 703 participants (631 with no SOGICE exposure and 72 with SOGICE exposure), ensuring that the dataset retained consistent values across key outcome variables while maintaining robust representation for the primary health outcomes analyzed.

We used a pairwise-like deletion approach for each regression model, in which each model retained as much data as possible by handling missing data separately for each outcome variable (eFigure 2 in Supplement 1 has a flowchart illustrating sample selection and stratification). Although this approach leads to varying sample sizes across models, it maximized available data for each analysis, particularly for diastolic and systolic blood pressure (n = 649), SI index (n = 548), and self-reported diagnosis of hypertension or high blood pressure (n = 688). All covariates and exposure measures were complete without missing data, minimizing the risk of bias due to missingness in model factors.

### Conversion Therapy Exposure

To assess SOGICE exposure, we modified an existing series of questions previously used in the 2019 Sex Now survey in Canada.^8,37^ SOGICE exposure was operationalized as self-reported responses to the following question: “Have you or any person with authority (parent, caregiver, counselor, community leader, etc.) ever tried to change your sexual orientation or gender identity?” Response options were (1) yes, sexual orientation; (2) yes, gender identity; or (3) no. Participants were able to select all. Responses were categorized as 0 (no exposure) for individuals who reported no history of such practices (reference group) or 1 (exposure) for those who reported yes to the SOGICE question for either option. Additionally, we asked participants how long they experienced SOGICE. Responses were categorized as 0 (no exposure) for individuals who reported no history of exposure (reference group) and 1 (≤1 year of exposure), or 2 (>1 year of exposure) for individuals who reported having undergone SOGICE. Additionally, participants were asked about age at first and last exposure, who tried to change their sexual orientation or gender identity, what types of SOGICE they were exposed to, and how these experiences affected them. The full questionnaire is available in the eAppendix in Supplement 1.

### Systemic Inflammation

Venous blood samples were collected at each visit. Level of SI was assessed by measuring plasma levels of C-reactive protein, interleukin (IL) 6, IL-10, and tumor necrosis factor α using an electrochemiluminescence immunoassay platform (MESO QuickPlex SQ 120MM [MESO Scale Diagnostics LLC]). Although IL-10 serves an anti-inflammatory function, it positively covaries with other inflammatory markers because when concentrations of proinflammatory proteins (C-reactive protein, IL-6, and tumor necrosis factor α) are elevated, IL-10 is upregulated as a compensatory mechanism to balance the inflammatory response and limit tissue damage.^44^ These biomarkers were chosen for their established relevance in signaling peripheral inflammatory responses.^44,45^ Following other studies,^46,47,48,49^ we calculated a composite index of inflammation by standardizing and summing the measures of all 4 markers to capture a holistic view of inflammatory activation.

### Blood Pressure

Both diastolic blood pressure (DBP) and systolic blood pressure (SBP) were measured using an automated blood pressure monitor (model BP5100 [OMRON Healthcare Inc]). Participants were seated comfortably, and measurements were taken after a 5-minute rest period, following best practices for blood pressure collection.^50^ For the purposes of this analysis, we use the most recently available measures of participants’ DBP and SBP collected at the same visit that SOGICE exposure was assessed.

### Self-Reported Diagnosis of HHBP

Secondary outcomes included self-reported diagnosis of HHBP. Participants were asked whether they “had ever been diagnosed with high blood pressure or hypertension by a health care provider,” with the following response option: no, yes, or do not know. Responses were transformed into binary variables that indicated the presence or absence of HHBP, with participants who reported “don’t know” dropped from analyses.

### Covariates

Data on age, gender identity, sexual orientation, race and ethnicity, educational level, body mass index (BMI), HIV status, and tobacco use were collected. Self-reported race and ethnicity were collected as part of the larger cohort study to better understand syndemic factors shaping the HIV epidemic in Chicago. The race and ethnicity categories we reported were Black or African American, Hispanic or Latinx, White, and other (including American Indian or Alaska Native, Asian, Native Hawaiian or Other Pacific Islander, multiracial, and/or other). Height and weight were assessed with a body composition analyzer with ultrasonic height measurement (mBCA 554, seca). BMI was calculated as weight in kilograms divided by height in meters squared at the most recent visit. HIV testing was performed at each visit for participants without a prior HIV diagnosis using a fourth-generation point-of-care test with a fingerstick blood sample (Alere Determine HIV1/2 Ab/Ag Combo [Abbott Laboratories]). Reactive results were confirmed with supplemental laboratory-based testing following Centers for Disease Control and Prevention guidelines.^51^ Tobacco use was evaluated through single-item questions on cigarette consumption (never, once or twice, occasionally but not regularly, regularly in the past, and regularly now).

### Statistical Analysis

Data analysis was performed using generalized linear models in the lme4 package^52^ to investigate the association between SOGICE exposure and our outcomes of interest: DBP, SBP, SI, and self-reported HHBP. Separate models were constructed for each outcome variable. Initial unadjusted models estimated the main association between SOGICE exposure and the outcomes without covariates. Two adjusted regression models were subsequently built: model A adjusted for age, gender identity, sexual orientation, and race and ethnicity (covariates), and model B adjusted for age, gender identity, sexual orientation, race and ethnicity, educational level, BMI, HIV status, and tobacco use (additional covariates). Results are reported as β coefficients and 95% CIs for models of DBP, SBP, and SI. For the association between SOGICE exposure and odds of self-reported HHBP, we used binomial logistic regression, following a modeling approach similar to the aforementioned. Results for these models are reported as odds ratios (ORs), 95% CIs, and SEs. Lastly, we replicated the aforementioned analysis substituting SOGICE exposure with SOGICE duration to conduct a dose-response analysis of the association between SOGICE and cardiovascular health. We also performed a sensitivity analysis on participants who reported exposure to SOCE (n = 49) and cardiovascular health because this group represents most of our sample. All statistical analyses were executed in R software version 4.4.1 (R Foundation for Statistical Computing),^53^ and the criterion for statistical significance was set at a 2-sided *P* < .05.

## Results

A total of 703 young adults who were AMAB (mean [SD] age, 26.75 [4.60] years) participated in the study. Table 1 provides an overview of the study sample. Of the 703 participants, 540 (76.8%) identified as cisgender and 163 (23.2%) as transgender or gender diverse. A total of 417 (59.3%) were gay, 162 (23.0%) bisexual or pansexual, and 124 (17.6%) other sexual identities. A total of 195 (27.7%) were Black or African American, 237 (33.7%) Hispanic or Latinx, 192 (27.3%) White, and 79 (11.2%) other race or ethnicity. Within our study sample, 631 participants (89.8%) reported no exposure to SOGICE, whereas 72 (10.2%) reported exposure to SOGICE. Among those who reported SOGICE exposure, 49 (7.0%) reported exposure to SOCE, 7 (1.0%) reported exposure to gender identity change efforts, and 16 (2.2%) reported exposure to SOGICE. Mean (SD) age of first SOGICE exposure was 12.79 (5.60) years, whereas mean (SD) age of last SOGICE exposure was 16.51 (4.82) years. Additionally, 42 participants (58.3%) reported 1 year or less of SOGICE exposure, and 30 (41.7%) reported more than 1 year of SOGICE exposure. Regarding sources of exposure, 39 participants (54.2%) reported exposure due to a parent, 27 (37.5%) due to their own choice, 4 (5.6%) due to another source (eg, grandparent), 1 (1.4%) due to a counselor, and 1 (1.4%) due to a community leader. Table 2 provides an overview of SOGICE-related characteristics among study sample participants.

**Table 1.** Characteristics of the Study Sample Stratified by SOGICE Exposure

Characteristic	No. (%) of participants^a^			*P* value^b^
	Overall (N = 703)	No SOGICE exposure (n = 631)	SOGICE exposure (n = 72)	
Age, y				
Mean (SD)	26.750 (4.6)	26.81 (4.57)	26.19 (4.85)	.28
Median (range)	27.00 (16.00 to 40.00)	27.00 (16.00 to 40.00)	26.00 (18.00 to 37.00)	
Gender identity				
Cisgender	540 (76.8)	495 (78.4)	45 (62.5)	.004
Transgender or gender diverse	163 (23.2)	136 (21.6)	27 (37.5)	
Sexual orientation				
Gay	417 (59.3)	378 (59.9)	39 (54.2)	.052
Bisexual or pansexual	162 (23.0)	149 (23.6)	13 (18.1)	
Other sexual identities	124 (17.6)	104 (16.5)	20 (27.8)	
Race and ethnicity				
Black or African American	195 (27.7)	173 (27.4)	22 (30.6)	.87
Hispanic or Latinx	237 (33.7)	215 (34.1)	22 (30.6)	
White	192 (27.3)	171 (27.1)	21 (29.2)	
Other^c^	79 (11.2)	72 (11.4)	7 (9.7)	
Educational level				
College graduate	253 (36.0)	231 (36.6)	22 (30.6)	.36
High school graduate or GED	160 (22.8)	146 (23.1)	14 (19.4)	
Some college	253 (36.0)	223 (35.3)	30 (41.7)	
Some high school or less	37 (5.3)	31 (4.9)	6 (8.3)	
BMI categories				
0 to 18.5	16 (2.3)	13 (2.1)	3 (4.2)	.24
>18.5 to 25.0	314 (44.9)	277 (44.2)	37 (51.4)	
>25.0 to 30.0	186 (26.6)	173 (27.6)	13 (18.1)	
>30.0 to 99.0	183 (26.2)	164 (26.2)	19 (26.4)	
Missing	4	4	0	
Diastolic blood pressure, mm Hg				
Mean (SD)	80.69 (11.13)	80.352 (10.70)	83.81 (14.16)	.02
Median (range)	80.00 (52.00 to 133.00)	80.00 (52.00 to 110.00)	81.00 (57.00 to 133.00)	
Missing	54	46	8	
Systolic blood pressure, mm Hg				
Mean (SD)	129.52 (14.00)	129.10 (13.59)	133.38 (16.77)	.02
Median (range)	129.00 (92.00 to 187.00)	128.00 (92.00 to 168.00)	132.50 (97.00 to 187.00)	
Missing	54	46	8	
Systemic inflammation index^d^				
Mean (SD)	−0.21 (2.29)	−0.29 (2.23)	0.54 (2.78)	.01
Median (range)	0.25 (−6.73 to 6.68)	0.28 (−6.73 to 5.95)	0.06 (−4.09 to 6.68)	
Missing	155	134	21	
HIV status				
Negative	560 (79.7)	501 (79.4)	59 (81.9)	.72
Positive	143 (20.3)	130 (20.6)	13 (18.1)	
Have you ever smoked cigarettes?				
Never	293 (41.7)	270 (42.8)	23 (31.9)	.40
Occasionally but not regularly	123 (17.5)	110 (17.4)	13 (18.1)	
Once or twice	124 (17.6)	110 (17.4)	14 (19.4)	
Regularly in the past	95 (13.5)	83 (13.2)	12 (16.7)	
Regularly now	68 (9.7)	58 (9.2)	10 (13.9)	
PROMIS depression T score^e^				
Mean (SD)	51.09 (9.83)	50.797 (9.82)	53.64 (9.57)	.02
Median (range)	52.10 (38.20 to 81.30)	52.10 (38.20 to 81.30)	55.50 (38.20 to 75.00)	
Diagnosed with high blood pressure or hypertension?				
No	631 (91.7)	572 (92.9)	59 (81.9)	.003
Yes	57 (8.3)	44 (7.1)	13 (18.1)	
Missing	15	15	0	

Abbreviations: BMI, body mass index (calculated as weight in kilograms divided by height in meters squared); GED, General Educational Development; PROMIS, Patient-Reported Outcomes Measurement Information System; SOGICE, sexual orientation and gender identity change efforts.

^a^ Unless otherwise indicated.

^b^ Two-sample *t* test or Pearson χ^2^ test.

^c^ Other races include American Indian or Alaska Native, Asian, Native Hawaiian or Other Pacific Islander, multiracial, and/or other.

^d^ Systemic inflammation index is the sum of *z* scores for C-reactive protein, interleukin (IL) 6, IL-10, and tumor necrosis factor α; range: −6.73 to 6.68; higher score indicates higher inflammation.

^e^ PROMIS depression T score for all items has a range from 38.2 to 81.3; higher score indicates higher depression.

**Table 2.** Characteristics of Study Sample Exposed to SOGICE

Characteristic	Participants, No. (%) (N = 72)^a^
Sexual orientation change efforts	
No	7 (9.7)
Yes	65 (90.3)
Gender identity change efforts	
No	49 (68.1)
Yes	23 (31.9)
SOGICE exposure	
No exposure	0
GICE exposure	7 (9.7)
SOCE exposure	49 (68.1)
SOGICE exposure	16 (22.2)
Who tried to change your sexual orientation or gender identity?	
No exposure	0
Your choice	27 (37.5)
Parent/caregiver	39 (54.2)
Counselor	1 (1.4)
Community leader	1 (1.4)
Other	4 (5.6)
Have you ever been exposed to any of the following conversion efforts?	
No exposure	0
Licensed health professional	13 (18.1)
Unlicensed counselor	3 (4.2)
Camp	1 (1.4)
Faith-based organization	7 (9.7)
Religious leader	6 (8.3)
Another religious individual	2 (2.8)
Other conversion efforts	7 (9.7)
None of the above	33 (45.8)
At what age [in years] did you first experience conversion efforts?	
Mean (SD)	12.79 (5.60)
Median (range)	14.00 (0.00-30.00)
At what age [in years] did you last experience conversion efforts?	
Mean (SD)	16.51 (4.82)
Median (range)	17.00 (1.00-30.00)
Missing	2 (2.8)
How many times did you experience conversion efforts?	
1	20 (27.78)
2	13 (18.06)
3	10 (13.89)
4	3 (4.17)
5	26 (36.11)
SOGICE exposure group	
No exposure	0
≤1 y of exposure	42 (58.33)
>1 y of exposure	30 (41.67)
How much did these experiences with conversion efforts affect you?	
No exposure	0
Extremely negatively	19 (26.4)
Extremely positively	9 (12.5)
Somewhat positively	8 (11.1)
Very negatively	35 (48.6)
Very positively	1 (1.4)

Abbreviations: GICE, gender identity change efforts; SOCE, sexual orientation change efforts; SOGICE, sexual orientation and gender identity change efforts.

^a^ Unless otherwise indicated.

In unadjusted regression models (Table 3), SOGICE exposure was associated with a 3.46–mm Hg higher DBP (95% CI, 0.60-6.32 mm Hg; *P* = .02), a 4.28–mm Hg higher SBP (95% CI, 0.68-7.87; *P* = .02), and a 0.83-point higher SI composite score (95% CI, 0.17-1.49; *P* = .02). In models adjusted for sociodemographic covariates, the associations remained significant for DBP (+3.58 mm Hg; 95% CI, 0.71-6.45 mm Hg; *P* = .02), SBP (+4.45 mm Hg; 95% CI, 0.82-8.09 mm Hg; *P* = .02), and SI (+0.72 points; 95% CI, 0.06-1.38 points; *P* = .03). After further adjusting for BMI, HIV status, and tobacco use, associations remained significant for DBP (+4.11 mm Hg; 95% CI, 1.37-6.86 mm Hg; *P* = .004), SBP (+5.34 mm Hg; 95% CI, 2.04-8.63 mm Hg; *P* = .002), and SI (+0.70 points; 95% CI, 0.08-1.32 points; *P* = .03). In binomial logistic regression models, SOGICE exposure was significantly associated with higher odds of self-reported HHBP (unadjusted model: odds ratio [OR], 2.86; 95% CI, 2.19-3.54; *P* = .003; adjusted model A: OR, 2.71; 95% CI, 2.01-3.41; *P* = .005; adjusted model B: OR, 2.98; 95% CI, 2.22-3.74; *P* = .005) (Table 3). A sensitivity analysis restricted to participants exposed to SOCE (n = 49) showed that the associations between SOCE and cardiovascular disease risk outcomes remained significant: DBP was estimated to be 4.08 mm Hg higher (95% CI, 1.20-6.95 mm Hg; *P* = .006), SBP was estimated to be 4.80 mm Hg higher (95% CI, 1.34-8.26 mm Hg; *P* = .007), SI was estimated to be 0.82 points higher (95% CI, 0.17-1.47 points; *P* = .01), and odds of self-reported HHBP were estimated to be 2.61 times higher (95% CI, 1.81-3.41; *P* = .02) compared with nonexposed participants (eTable and eFigure 3 in Supplement 1).

**Table 3.** Models of Cardiovascular Health for the RADAR Study Sample

Characteristic	β (95% CI)											
	Diastolic blood pressure			Systolic blood pressure			Systemic inflammation			Diagnosed with hypertension or high blood pressure		
	Unadjusted model	Adjusted model A	Normal, adjusted model B	Normal, unadjusted model	Normal, adjusted model A	Normal, adjusted model B	Logistic, unadjusted model	Adjusted model A	Adjusted model B	Unadjusted model	Adjusted model A	Adjusted model B
Conversion therapy exposure: yes (reference: no)	3.46 (0.60 to 6.32)	3.58 (0.71 to 6.45)	4.11 (1.37 to 6.86)	4.28 (0.68 to 7.87)	4.45 (0.82 to 8.09)	5.34 (2.04 to 8.63)	0.83 (0.17 to 1.49)	0.72 (0.06 to 1.38)	0.70 (0.08 to 1.32)	2.86 (2.19 to 3.54)	2.71 (2.01 to 3.41)	2.98 (2.22 to 3.74)
*P* value	.02	.02	.004	.02	.02	.002	.02	.03	.03	.003	.005	.005
Age	NA	0.29 (0.10 to 0.48)	0.17 (−0.04 to 0.37)	NA	0.18 (−0.06 to 0.43)	−0.03 (−0.28 to 0.22)	NA	0.05 (0.01 to 0.10)	0.01 (−0.04 to 0.06)	NA	1.07 (1.01 to 1.13)	1.06 (0.99 to 1.13)
*P* value	NA	.003	.12	NA	.14	.80	NA	.03	.63	NA	.04	.11
Transgender (reference: cisgender)	NA	0.68 (−1.61 to 2.97)	−0.55 (−2.77 to 1.68)	NA	−0.91 (−3.81 to 1.99)	−2.66 (−5.33 to 0.01)	NA	0.30 (−0.22 to 0.82)	0.14 (−0.36 to 0.64)	NA	1.93 (1.23 to 2.62)	1.34 (0.59 to 2.09)
*P* value	NA	.56	.63	NA	.54	.052	NA	.26	.58	NA	.06	.45
Bisexual or pansexual (reference: lesbian or gay)	NA	0.06 (−2.09 to 2.21)	−0.16 (−2.24 to 1.92)	NA	−0.41 (−3.13 to 2.32)	−0.75 (−3.24 to 1.75)	NA	−0.07 (−0.56 to 0.42)	−0.17 (−0.64 to 0.29)	NA	0.76 (−0.01 to 1.53)	0.68 (−0.12 to 1.47)
*P* value	NA	.96	.88	NA	.77	.56	NA	.78	.46	NA	.48	.34
Other sexual identities	NA	−0.39 (−2.10 to 2.21)	−0.50 (−3.01 to 2.02)	NA	0.99 (−2.30 to 4.28)	0.57 (−2.45 to 3.59)	NA	−0.06 (−0.64 to 0.53)	0.02 (−0.54 to 0.58)	NA	0.93 (0.14 to 1.72)	1.07 (0.22 to 1.92)
*P* value	NA	.77	.70	NA	.56	.71	NA	.85	.94	NA	.85	.87
Black or African American (reference: White)	NA	1.38 (−1.02 to 3.77)	2.14 (−0.42 to 4.69)	NA	−0.12 (−3.14 to 2.91)	0.79 (−2.28 to 3.85)	NA	0.37 (−0.16 to 0.91)	0.04 (−0.53 to 0.62)	NA	0.71 (−0.08 to 1.50)	0.50 (−0.45 to 1.45)
*P* value	NA	.26	.10	NA	.94	.62	NA	.17	.88	NA	.40	.15
Hispanic or Latinx	NA	1.18 (−1.04 to 3.40)	0.79 (−1.37 to 2.96)	NA	−0.01 (−2.82 to 2.79)	−0.94 (−3.54 to 1.65)	NA	0.15 (−0.36 to 0.65)	−0.20 (−0.69 to 0.29)	NA	1.11 (0.41 to 1.81)	0.80 (0.05 to 1.56)
*P* value	NA	.30	.47	NA	.99	.48	NA	.56	.43	NA	.77	.58
Other race or ethnicity^a^	NA	−0.05 (−3.14 to 3.05)	1.27 (−1.76 to 4.30)	NA	−1.40 (−5.32 to 2.51)	0.89 (−2.75 to 4.52)	NA	−0.53 (−1.22 to 0.17)	−0.60 (−1.27 to 0.08)	NA	0.92 (−0.09 to 1.92)	0.87 (−0.25 to 1.99)
*P* value	NA	.98	.41	NA	.48	.63	NA	.14	.09	NA	.87	.81
High school graduate or GED (reference: college graduate)	NA	NA	−1.01 (−3.43 to 1.41)	NA	NA	−0.80 (−3.71 to 2.10)	NA	NA	−0.03 (−0.57 to 0.50)	NA	NA	1.29 (0.38 to 2.20)
*P* value	NA	NA	.41	NA	NA	.59	NA	NA	.90	NA	NA	.58
Some college	NA	NA	−0.69 (−2.79 to 1.41)	NA	NA	−0.24 (−2.76 to 2.28)	NA	NA	0.22 (−0.23 to 0.67)	NA	NA	1.64 (0.92 to 2.36)
*P* value	NA	NA	.52	NA	NA	.85	NA	NA	.34	NA	NA	.18
Some high school or less	NA	NA	−0.35 (−4.30 to 3.61)	NA	NA	1.15 (−3.60 to 5.90)	NA	NA	0.44 (−0.44 to 1.31)	NA	NA	1.60 (0.16 to 3.04)
*P* value	NA	NA	.86	NA	NA	.64	NA	NA	.33	NA	NA	.52
BMI <18.5	NA	NA	2.38 (−2.97 to 7.74)	NA	NA	−2.87 (−9.30 to 3.57)	NA	NA	0.09 (−1.22 to 1.40)	NA	NA	2.18 (−0.02 to 4.38)
*P* value	NA	NA	.38	NA	NA	.38	NA	NA	.90	NA	NA	.49
BMI 25-30	NA	NA	5.12 (3.12 to 7.13)	NA	NA	9.32 (6.91 to 11.73)	NA	NA	0.64 (0.21 to 1.08)	NA	NA	2.00 (1.09 to 2.90)
*P* value	NA	NA	<.001	NA	NA	<.001	NA	NA	.005	NA	NA	.13
BMI >30	NA	NA	8.89 (6.85 to 10.93)	NA	NA	14.52 (12.06 to 16.97)	NA	NA	1.86 (1.41 to 2.31)	NA	NA	7.54 (6.76 to 8.31)
*P* value	NA	NA	<.001	NA	NA	<.001	NA	NA	<.001	NA	NA	<.001
HIV status positive (reference: negative)	NA	NA	−1.28 (−3.61 to 1.06)	NA	NA	−1.50 (−4.31 to 1.30)	NA	NA	0.81 (0.33 to 1.30)	NA	NA	0.88 (0.01 to 1.76)
*P* value	NA	NA	.28	NA	NA	.29	NA	NA	.002	NA	NA	.78
Have you ever smoked cigarettes? Occasionally but not regularly (reference: never)	NA	NA	−1.04 (−3.40 to 1.32)	NA	NA	−0.59 (−3.43 to 2.24)	NA	NA	0.48 (−0.06 to 1.03)	NA	NA	1.23 (0.39 to 2.07)
*P* value	NA	NA	.39	NA	NA	.68	NA	NA	.08	NA	NA	.63
Have you ever smoked cigarettes? Once or twice	NA	NA	−0.73 (−3.06 to 1.60)	NA	NA	−0.34 (−3.19 to 2.41)	NA	NA	0.44 (−0.08 to 0.96)	NA	NA	0.82 (−0.04 to 1.68)
*P* value	NA	NA	.54	NA	NA	.79	NA	NA	.10	NA	NA	.65
Have you ever smoked cigarettes? Regularly in the past	NA	NA	−0.86 (−3.48 to 1.76)	NA	NA	0.70 (−2.45 to 3.84)	NA	NA	0.14 (−0.42 to 0.69)	NA	NA	0.73 (−0.28 to 1.74)
*P* value	NA	NA	.52	NA	NA	.66	NA	NA	.64	NA	NA	.54
Have you ever smoked cigarettes? Regularly now	NA	NA	0.68 (−2.35 to 3.70)	NA	NA	2.05 (−1.58 to 5.68)	NA	NA	0.80 (0.15 to 1.44)	NA	NA	1.26 (0.29 to 2.23)
*P* value	NA	NA	.66	NA	NA	.27	NA	NA	.02	NA	NA	.64
Constant	80.35 (79.45 to 81.25)	71.63 (66.38 to 76.88)	72.47 (66.56 to 78.38)	129.10 (127.10 to 130.23)	124.51 (117.87 to 131.15)	124.72 (117.62 to 131.83)	−0.29 (−0.49 to −0.09)	−1.94 (−3.33 to −0.55)	−1.73 (−3.16 to −0.30)	0.08 (−0.23 to 0.38)	0.01 (−1.77 to 1.79)	0.01 (−2.11 to 2.12)
*P* value	<.001	<.001	<.001	<.001	<.001	<.001	.005	.007	.0	<.001	<.001	<.001
Observations	649	649	648	649	649	648	548	548	547	688	688	684
Log likelihood	−2482.18	−2475.44	−2430.99	−2630.31	−2628.25	−2549.92	−1230.04	−1222.70	−1178.01	−192.51	−187.97	−168.47
Akaike information criterion	4968.37	4968.88	4901.98	5264.61	5274.50	5139.84	2464.07	2463.40	2396.02	389.02	393.95	376.94

Abbreviations: BMI, body mass index (calculated as weight in kilograms divided by height in meters squared); GED, General Educational Development; NA, not applicable.

^a^ Other races include American Indian or Alaska Native, Asian, Native Hawaiian or Other Pacific Islander, multiracial, and/or other.

Finally, there was a significant association between longer exposure to SOGICE and cardiovascular disease risk (Table 4 and Figure). Individuals exposed to more than 1 year of SOGICE demonstrated significantly higher DBP (+6.36 mm Hg; 95% CI, 1.36-11.35 mm Hg; *P* = .01) and SBP (+4.57 mm Hg; 95% CI, 0.41-8.73 mm Hg; *P* = .03) compared with those with 1 year or less and no exposure. Moreover, those exposed for 1 year or less had significantly higher DBP (+4.63 mm Hg; 95% CI, 0.43-8.83 mm Hg; *P* = .03) and SBP (+3.79 mm Hg; 95% CI, 0.30-7.29 mm Hg; *P* = .03) relative to individuals with no exposure. In addition, SI was significantly elevated among individuals reporting more than 1 year of SOGICE exposure (+1.27 points; 95% CI, 0.30-2.23 points; *P* = .01), indicating a heightened inflammatory response associated with prolonged exposure. However, those with less than 1 year of exposure did not show increased SI compared with individuals without SOGICE exposure. Additionally, the odds of self-reported HHBP were significantly higher for individuals with more than 1 year of exposure (OR, 3.24; 95% CI, 2.17-4.31; *P* = .03) and for those with 1 year or less of exposure (OR, 2.79; 95% CI, 1.80-3.78; *P* = .04).

**Table 4.** Dose-Response Regression Models for SOGICE Exposure for Study Sample RADAR Participants

	β (95% CI)				
	Diastolic blood pressure	Systolic blood pressure	Systemic inflammation	Diagnosed with hypertension or high blood pressure
SOGICE exposure of ≤1 y (reference: no exposure)	4.63 (0.43 to 8.83)	3.79 (0.30 to 7.29)	0.33 (−0.46 to 1.12)	2.79 (1.80 to 3.78)
*P* value	.03	.03	.41	.04
SOGICE exposure of >1 y (reference: no exposure)	6.36 (1.36 to 11.35)	4.57 (0.41 to 8.73)	1.27 (0.30 to 2.23)	3.24 (2.17 to 4.31)
*P* value	.01	.03	.01	.03
Age	−0.04 (−0.28 to 0.21)	0.16 (−0.04 to 0.37)	0.01 (−0.04 to 0.06)	1.06 (0.99 to 1.13)
*P* value	.79	.12	.69	.11
Transgender (reference: cisgender)	−2.61 (−5.29 to 0.07)	−0.53 (−2.76 to 1.70)	0.19 (−0.32 to 0.69)	1.35 (0.60 to 2.11)
*P* value	.057	.64	.47	.43
Bisexual or pansexual (reference: lesbian or gay)	−0.75 (−3.25 to 1.74)	−0.16 (−2.24 to 1.92)	−0.18 (−0.65 to 0.28)	0.67 (−0.13 to 1.47)
*P* value	.56	.88	.45	.33
Other sexual identities	0.48 (−2.56 to 3.52)	−0.54 (−3.07 to 1.99)	−0.02 (−0.58 to 0.54)	1.06 (0.20 to 1.92)
*P* value	.76	.68	.94	.90
Straight or heterosexual	0.83 (−2.24 to 3.90)	2.15 (−0.40 to 4.71)	0.08 (−0.50 to 0.65)	0.50 (−0.45 to 1.46)
*P* value	.60	.10	.79	.16
Black or African American (reference: White)	−0.94 (−3.54 to 1.65)	0.79 (−1.37 to 2.96)	−0.18 (−0.67 to 0.31)	0.80 (0.04 to 1.56)
*P* value	.48	.47	.48	.58
Hispanic or Latinx	0.90 (−2.74 to 4.53)	1.27 (−1.76 to 4.30)	−0.59 (−1.26 to 0.09)	0.87 (−0.25 to 1.98)
*P* value	.63	.41	.09	.80
Other race or ethnicity	−0.82 (−3.73 to 2.09)	−1.02 (−3.44 to 1.40)	−0.03 (−0.57 to 0.50)	1.29 (0.38 to 2.20)
*P* value	.58	.41	.90	.58
High school graduate or GED (reference: college graduate)	−0.26 (−2.79 to 2.26)	−0.70 (−2.80 to 1.40)	0.22 (−0.23 to 0.67)	1.63 (0.91 to 2.36)
*P* value	.84	.51	.34	.18
Some college	1.20 (−3.56 to 5.95)	−0.33 (−4.29 to 3.63)	0.49 (−0.39 to 1.36)	1.61 (0.16 to 3.05)
*P* value	.62	.87	.28	.52
Some high school or less	−2.98 (−9.43 to 3.48)	2.34 (−3.03 to 7.71)	−0.02 (−1.34 to 1.30)	2.14 (−0.07 to 4.35)
*P* value	.367	.40	.98	.500
BMI <18.5	9.29 (6.88 to 11.71)	5.11 (3.10 to 7.12)	0.64 (0.20 to 1.07)	1.99 (1.08 to 2.89)
*P* value	<.001	<.001	.005	.14
BMI 25-30	14.51 (12.06 to 16.97)	8.89 (6.84 to 10.93)	1.86 (1.41 to 2.30)	7.53 (6.75 to 8.30)
*P* value	<.001	<.001	<.001	<.001
BMI >30	−1.48 (−4.29 to 1.32)	−1.27 (−3.60 to 1.07)	0.81 (0.33 to 1.30)	0.89 (0.01 to 1.76)
*P* value	.30	.29	.002	.79
HIV status positive (reference: negative)	−0.56 (−3.40 to 2.28)	−1.02 (−3.39 to 1.34)	0.48 (−0.06 to 1.03)	1.24 (0.40 to 2.08)
*P* value	.70	.40	.08	.62
Have you ever smoked cigarettes? Occasionally but not regularly (reference: never)	−0.35 (−3.15 to 2.46)	−0.71 (−3.05 to 1.62)	0.47 (−0.05 to 0.99)	0.83 (−0.03 to 1.69)
*P* value	.81	.55	.08	.67
Have you ever smoked cigarettes? Once or twice	0.71 (−2.44 to 3.86)	−0.86 (−3.48 to 1.77)	0.12 (−0.43 to 0.68)	0.73 (−0.28 to 1.74)
*P* value	.66	.52	.66	.55
Have you ever smoked cigarettes? Regularly in the past	2.07 (−1.57 to 5.70)	0.68 (−2.34 to 3.71)	0.78 (0.13 to 1.43)	1.26 (0.28 to 2.23)
*P* value	.27	.66	.02	.65
Constant	124.78 (117.67 to 131.88)	72.50 (66.58 to 78.41)	−1.69 (−3.12 to −0.26)	0.01 (−2.11 to 2.12)
*P* value	<.001	<.001	.02	<.001
Observations	648	648	547	684
Log likelihood	−2549.78	−2430.94	−1176.85	−168.45
Akaike information criterion	5141.55	4903.89	2395.70	378.90

Abbreviations: BMI, body mass index (calculated as weight in kilograms divided by height in meters squared); GED, General Educational Development; SOGICE, sexual orientation and gender identity change efforts.

**Figure.**
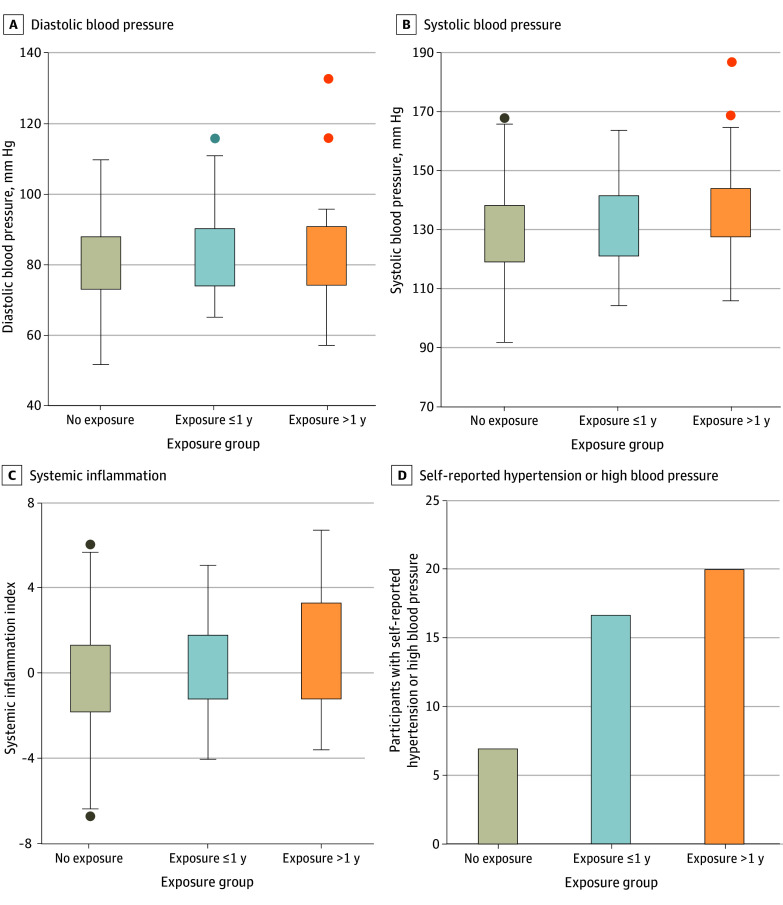
Dose Response of Sexual Orientation and Gender Identity Change Efforts (SOGICE) Exposure on Cardiovascular Health Measures In panels A, B, and C, error bars indicate SDs; solid circles, outliers; boxes, IQRs.

## Discussion

In this study, we used data from a diverse observational cohort of SGM young adults who were AMAB to test whether SOGICE exposure was associated with increased DBP, SBP, SI, and odds of self-reported HHBP, important measures of cardiovascular disease risk. We found that exposure to SOGICE was significantly associated with blood pressure and a multimarker index of SI. These associations persisted even after adjusting for a range of demographic, behavioral, and health-related covariates, which suggests that the associations of SOGICE exposure with cardiovascular disease risk are not explained by health behaviors or HIV status. Our findings suggest that exposure to SOGICE may be a potential risk factor for cardiovascular disease among SGM young adults. Moreover, we observed a dose response with duration of SOGICE exposure and reduced cardiovascular health. To our knowledge, this is the first study to investigate the association between SOGICE exposure and cardiovascular health among SGM young adults who were AMAB. Our results highlight the need for health care practitioners and policymakers to consider the harmful effects of SOGICE on physical health in their efforts to support SGM populations.

Our findings add to the increasing body of evidence documenting the deleterious health consequences of SOGICE among SGM young adults. Much of the current literature on SOGICE has documented how it is associated with increased risk for adverse mental health outcomes. For example, multiple studies^8,9,36^ have found that SOGICE increases depressive symptoms, loneliness, regular illicit drug use, suicidal ideation, and suicidal behavior and is associated with lower access to medical care. Our findings support and extend this by illustrating a deleterious association of SOGICE with physical health, specifically risk factors for cardiovascular disease.

There are several potential interrelated causal pathways through which exposure to SOGICE during key critical periods of growth and development^39,40,41^ may lead to adverse health outcomes in adulthood, specifically through stress-responsive biological mechanisms. Given that the mean first age of exposure to SOGICE in our sample occurred around puberty and early adolescence (13 years of age), which is a critical period of growth and development for SGM populations,^54,55,56,57^ it is possible that stress from SOGICE exposure during this period shapes the risk of adverse biological health outcomes later in adulthood. One possible biological pathway involves SOGICE triggering the hypothalamic-pituitary-adrenal axis, which regulates cortisol secretion, a pivotal steroidal coordinator of the physiologic stress responses.^25^ The chronic activation of this axis due to prolonged SOGICE exposure could lead to cortisol dysregulation, which has been linked to increased hypertension, dyslipidemia, and type 2 diabetes—all risk factors for cardiovascular diseases.^58,59^ Alternatively, chronic stress is linked to elevated levels of proinflammatory cytokines, such as IL-6 and tumor necrosis factor α,^34,60,61^ which are associated with increased risks of cardiovascular diseases, obesity, and metabolic syndrome.^45^ This ongoing inflammation and dysregulated stress response can (in tandem or separately) lead to autonomic nervous system imbalances, enhancing sympathetic activity and reducing parasympathetic tone, thereby increasing heart rate, blood pressure, and vascular hypertonicity.^62^ Lastly, epigenetic mechanisms may represent another pathway through which SOGICE exposure can shape stress reactivity and cardiovascular disease risk over time. Changes in DNA methylation can have lasting effects on immune function, metabolic regulation, and overall disease susceptibility,^49^ which can lead to increased risk of cardiovascular disease. Future research is needed to better characterize the biological pathways and mechanisms through which SOGICE exposure gets under the skin to shape physiologic function and the development of adverse cardiometabolic health outcomes in adulthood.^63^

### Implications for Health and Society

The association between SOGICE exposure and elevated cardiovascular disease risk has significant public health implications. Our findings suggest that SOGICE practices are associated with negative physical health outcomes. Although awareness of the harm caused by SOGICE is increasing, legal bans remain inconsistent across the US. Policymakers should consider these results when shaping laws to protect SGM individuals from further harm. Additionally, health care practitioners play a crucial role in the creation of affirming and supportive environments for SGM patients, particularly those with past SOGICE experiences. Future research should examine whether psychological interventions that promote healing from SOGICE can reverse the negative physical health outcomes included in our study. Public health programs focused on cardiovascular health should also recognize the stressors faced by SGM populations and offer targeted interventions,^31^ such as providing access to SGM-affirming mental health services, implementing stress-reduction programs tailored to SGM people and their families,^33,64^ and facilitating community-based support groups that address mental and physical health.^65^ Addressing the harmful effects of SOGICE will require coordinated efforts from health care professionals, policymakers, and public health leaders. By implementing policies that prohibit SOGICE and promoting acceptance of sexual and gender diversity, we can better support the health of SGM individuals.

### Limitations 

Although our study reveals important insights about the association between SOGICE exposure and increased blood pressure and SI among SGM young adults, there are limitations in need of discussion. First, the sample of participants who reported exposure to SOGICE may not be representative of the broader SGM community and/or capture the full spectrum of experiences among those exposed to SOGICE. Second, our reliance on self-reported data for SOGICE exposure and hypertension or high blood pressure diagnosis introduces potential biases. For instance, participants may underreport experiences related to SOGICE due to stigma or may not accurately remember past health details, which could influence the results. The fact that these data were collected in a sample of adolescents and young adults partially reduces concerns about recall bias, however.^66^ Survival bias may represent another limitation, given the potential for increased mortality (eg, suicidality)^7^ among those exposed to SOGICE, which may bias our analysis toward null results. Third, future research should aim to disentangle the effects of SOCE and gender identity change efforts with larger samples. Additionally, given the cross-sectional nature of our analysis, future longitudinal research is needed to establish temporal relationships and mechanisms between SOGICE and health. Fourth, although we adjusted for a variety of covariates, there may still be unmeasured factors we did not account for, such as the severity of SOGICE, other adverse childhood experiences, physical activity, income, the presence of supportive social networks, and/or other mental health conditions.

## Conclusions

In this cohort study of SGM young adults who were AMAB, exposure to SOGICE was associated with adverse cardiovascular health indicators, including elevated DBP and SBP, increased SI, and higher odds of self-reported HHBP. This is the first study, to our knowledge, to document elevated blood pressure and SI, important factors shaping the risk of adverse cardiovascular health outcomes, among SGM people exposed to SOGICE. Although our sample consists of young adults (mean age, 26.75 years), the detection of elevated blood pressure and SI suggests early manifestations of cardiovascular risk. This finding underscores the potential for these early markers to predict future cardiovascular disease, highlighting the importance of early intervention and continued longitudinal follow-up. Our findings support and extend the evidence illustrating a deleterious effect of SOGICE on psychosocial health. Our findings also support bans on SOGICE and enforcement of existing bans to eventually eliminate the adverse health consequences associated with these practices. Future research should aim to characterize the biological pathways through which exposure to SOGICE may impact health outcomes across the life course among SGM people.

## Notes

Supplement 1. **eFigure 1.** State Level Legislation of Banning or Restricting SOGICE in the United States **eFigure 2.** Flowchart Illustrating Sample Stratification **eFigure 3.** Sensitivity Analysis Forest Plot of SOCE and Cardiovascular Disease Risk **eAppendix.** Lifetime Conversion Therapy Exposure Questionnaire **eTable.** Sensitivity Analysis Regression Models for SOCE

Supplement 2.
Data Sharing Statement
