# Supplementary material for: Conversion Therapy Exposure and Elevated Cardiovascular Disease Risk
Source: JAMA Netw Open. 2025 May 6;8(5):e258745. doi: 10.1001/jamanetworkopen.2025.8745 (PMC12056572; doi:10.1001/jamanetworkopen.2025.8745)
Supplement: Supplement 1. — eFigure 1. State Level Legislation of Banning or Restricting SOGICE in the United States eFigure 2. Flowchart Illustrating Sample Stratification eFigure 3. Sensitivity Analysis Forest Plot of SOCE and Cardiovascular Disease Risk eAppendix. Lifetime Conversion Therapy Exposure Questionnaire eTable. Sensitivity Analysis Regression Models for SOCE [file jamanetwopen-e258745-s001.pdf]

Supplemental Online Content

Gibb JK, Schrock JM, Smith MS, D'Aquila RT, McDade TW, Mustanski B. Conversion therapy exposure and elevated cardiovascular disease risk. *JAMA Netw Open*. 2025;8(5):e258745. doi:10.1001/jamanetworkopen.2025.8745

eFigure 1. State Level Legislation of Banning or Resticting SOGICE in the United States
eFigure 2. Flow Chart Illustrating Sample Stratification
eFigure 3. Sensitivity Analysis Forest Plot of SOCE and Cardiovascular Disease Risk
eAppendix 1. Lifetime Conversion Therapy Exposure Questionnaire
eTable. Sensitivity Analysis Regression Models for SOCE

This supplemental material has been provided by the authors to give readers additional information about their work.

© 2025 Gibb JK et al. *JAMA Network Open*.

eFigure 1. State Level Legislation of Banning or Resticting SOGICE in the United States

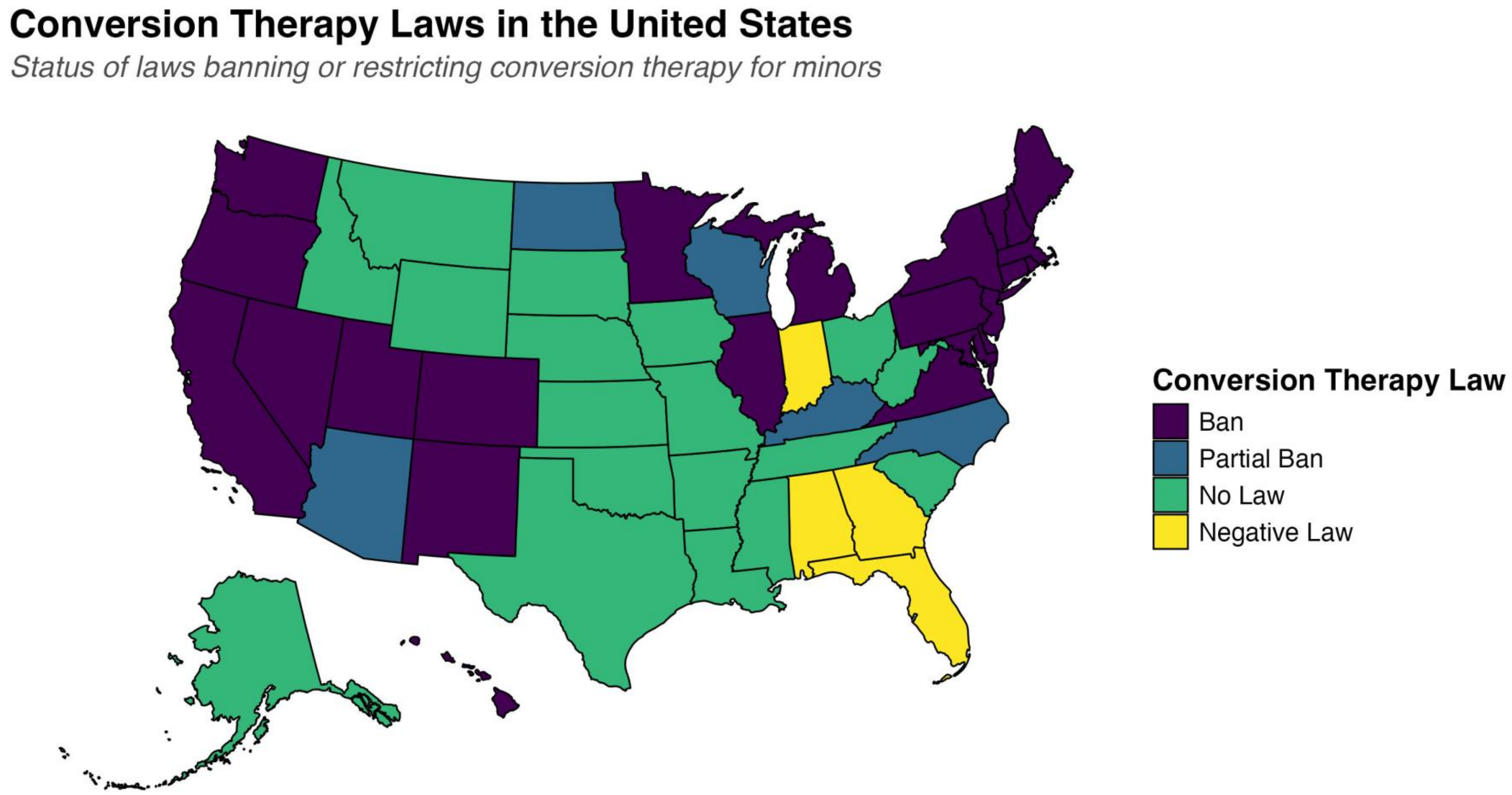

Source: Equality maps: conversion therapy laws: https://www.lgbtmap.org/equality-maps/conversion_therapy

© 2025 Gibb JK et al. *JAMA Network Open*.

eFigure 2. Flow Chart Illustrating Sample Stratification

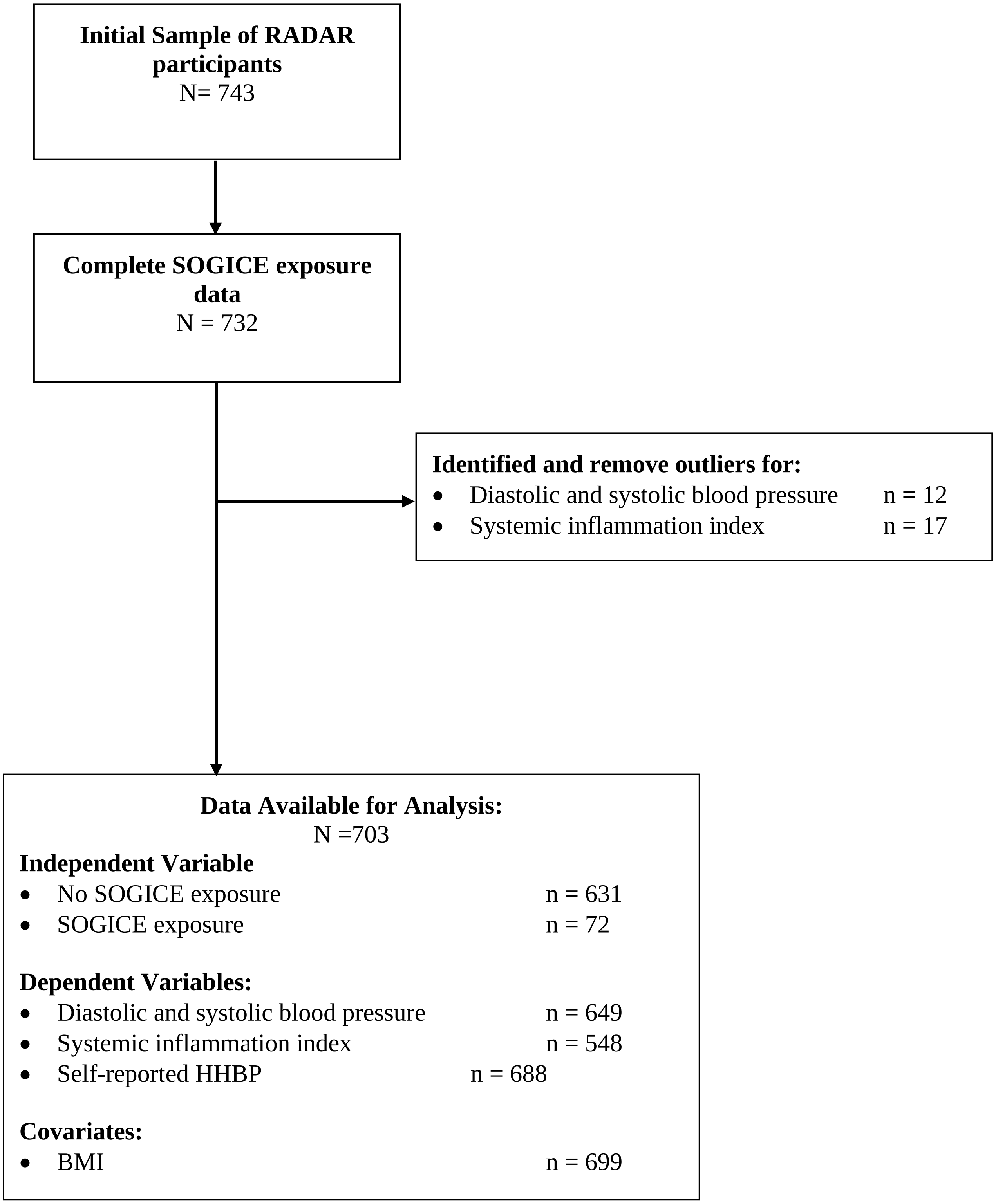

© 2025 Gibb JK et al. *JAMA Network Open*.

eTable. Sensitivity Analysis Regression Models for SOCE **Exposure**

	Dependent variable:			
	Diastolic Blood Pressure	Systolic Blood Pressure	Systemic Inflammation	Hypertension Diagnosis
	normal	*normal*	*normal*	*logistic*
	(1)	(2)	(3)	(4)
Sexual Orientation Change Efforts: Yes (ref: SOCE: No)	4.078	4.802	0.822	2.612
	(1.202, 6.953)	(1.343, 8.262)	(0.173, 1.471)	(1.811, 3.413)
	p = 0.006	p = 0.007	p = 0.014	p = 0.019
Age	0.158	-0.044	0.011	1.059
	(-0.050, 0.365)	(-0.293, 0.206)	(-0.039, 0.061)	(0.987, 1.132)
	p = 0.137	p = 0.732	p = 0.667	p = 0.118
Transgender (Ref. Cisgender)	-0.399	-2.444	0.162	1.425
	(-2.617, 1.818)	(-5.112, 0.224)	(-0.337, 0.662)	(0.681, 2.168)
	p = 0.725	p = 0.074	p = 0.525	p = 0.351
Bisexual/Pansexual (ref. Lesbian/Gay)	-0.201	-0.815	-0.174	0.656
	(-2.278, 1.877)	(-3.315, 1.685)	(-0.639, 0.292)	(-0.141, 1.452)
	p = 0.850	p = 0.524	p = 0.466	p = 0.300
Other Sexual Identities	-0.589	0.463	0.006	1.033
	(-3.105, 1.926)	(-2.563, 3.490)	(-0.555, 0.566)	(0.183, 1.883)
	p = 0.647	p = 0.765	p = 0.984	p = 0.942
Straight/Heterosexual	2.160	0.839	0.048	0.503
	(-0.392, 4.713)	(-2.232, 3.910)	(-0.522, 0.617)	(-0.445, 1.451)
	p = 0.098	p = 0.593	p = 0.871	p = 0.156

© 2025 Gibb JK et al. *JAMA Network Open*.

Black or African American (ref. White)	0.737<br>(-1.427, 2.902)<br>p = 0.505	-1.003<br>(-3.608, 1.601)<br>p = 0.451	-0.205<br>(-0.696, 0.286)<br>p = 0.414	0.792<br>(0.034, 1.549)<br>p = 0.546
Hispanic/Latinx	1.199<br>(-1.830, 4.227)<br>p = 0.439	0.809<br>(-2.835, 4.452)<br>p = 0.664	-0.599<br>(-1.275, 0.076)<br>p = 0.083	0.854<br>(-0.263, 1.970)<br>p = 0.782
Other Race/Ethnicities	-1.105<br>(-3.525, 1.316)<br>p = 0.372	-0.914<br>(-3.826, 1.999)<br>p = 0.539	-0.050<br>(-0.588, 0.488)<br>p = 0.856	1.264<br>(0.357, 2.171)<br>p = 0.614
High School/GED (ref. College Graduate)	-0.704<br>(-2.804, 1.395)<br>p = 0.512	-0.245<br>(-2.771, 2.281)<br>p = 0.850	0.215<br>(-0.236, 0.667)<br>p = 0.351	1.661<br>(0.941, 2.380)<br>p = 0.168
Some college	-0.215<br>(-4.167, 3.738)<br>p = 0.916	1.349<br>(-3.407, 6.105)<br>p = 0.579	0.443<br>(-0.431, 1.316)<br>p = 0.322	1.728<br>(0.306, 3.150)<br>p = 0.452
Some high school or less	2.607<br>(-2.750, 7.963)<br>p = 0.341	-2.569<br>(-9.014, 3.876)<br>p = 0.435	0.143<br>(-1.164, 1.451)<br>p = 0.831	2.429<br>(0.248, 4.609)<br>p = 0.426
BMI < 18.5	5.118<br>(3.111, 7.126)<br>p = 0.00000	9.296<br>(6.880, 11.711)<br>p = 0.000	0.651<br>(0.214, 1.087)<br>p = 0.004	1.969<br>(1.067, 2.870)<br>p = 0.141
BMI 25-30	8.889<br>(6.845, 10.933)<br>p = 0.000	14.504<br>(12.046, 16.963)<br>p = 0.000	1.860<br>(1.413, 2.307)<br>p = 0.000	7.444<br>(6.673, 8.214)<br>p = 0.00000
BMI > 30	-1.191<br>(-3.526, 1.144)	-1.417<br>(-4.227, 1.392)	0.823<br>(0.338, 1.308)	0.902<br>(0.031, 1.773)

© 2025 Gibb JK et al. *JAMA Network Open*.

	p = 0.318	p = 0.324	p = 0.001	p = 0.817
HIV Status: Positive (ref. Negative)	-1.033	-0.565	0.472	1.228
	(-3.394, 1.329)	(-3.407, 2.276)	(-0.073, 1.017)	(0.393, 2.063)
	p = 0.392	p = 0.697	p = 0.090	p = 0.631
Have you ever smoked cigarettes? Occasionally but not regularly (ref. Never)	-0.748	-0.389	0.434	0.824
	(-3.081, 1.585)	(-3.195, 2.417)	(-0.086, 0.955)	(-0.031, 1.680)
	p = 0.530	p = 0.787	p = 0.103	p = 0.659
Have you ever smoked cigarettes? Once or twice	-0.793	0.808	0.140	0.740
	(-3.411, 1.826)	(-2.342, 3.958)	(-0.417, 0.698)	(-0.264, 1.744)
	p = 0.554	p = 0.616	p = 0.622	p = 0.558
Have you ever smoked cigarettes? Regularly in the past	0.734	2.149	0.800	1.272
	(-2.291, 3.758)	(-1.490, 5.787)	(0.153, 1.447)	(0.306, 2.238)
	p = 0.635	p = 0.248	p = 0.016	p = 0.626
Constant	72.731	125.096	-1.695	0.006
	(66.825, 78.637)	(117.990, 132.202)	(-3.123, -0.267)	(-2.101, 2.113)
	p = 0.000	p = 0.000	p = 0.021	p = 0.00001
Observations	648	648	547	684
Log Likelihood	-2,431.450	-2,551.281	-1,177.338	-169.552
Akaike Inf. Crit.	4,902.900	5,142.562	2,394.675	379.105

© 2025 Gibb JK et al. *JAMA Network Open*.

eFigure 3. Sensitivity Analysis Forest Plot of SOCE and Cardiovascular Disease Risk

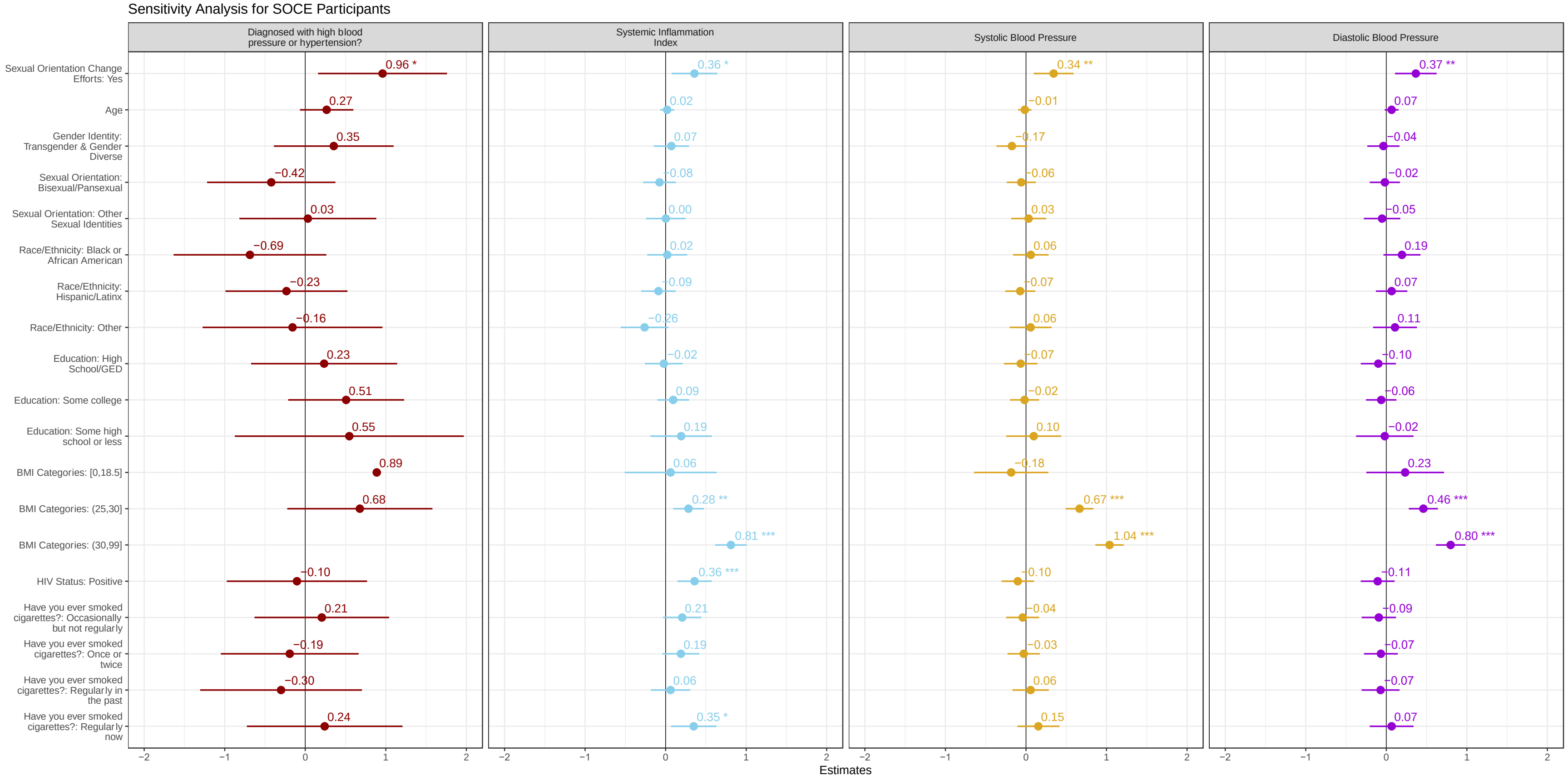

© 2025 Gibb JK et al. *JAMA Network Open*.

eAppendix. Lifetime Conversion Therapy Exposure Questionnaire

These questions are about your experiences with trying to change your sexual orientation or gender identity. The following questions are important but may be uncomfortable or triggering. They will ask you about your experiences with conversion therapy. Remember, you can skip any questions you prefer not to answer.

1. Have you or any person with authority (parent, caregiver, counselor, community leader, etc.) ever tried to change your sexual orientation or gender identity? (check all that apply)

   a) Yes, sexual orientation
   b) Yes, gender identity
   c) No

1a. [IF YES ABOVE to option a, ASK] Who tried to change your sexual orientation? (check all that apply)

   a) Your choice
   b) Parent
   c) Caregiver
   d) Counselor
   e) Community leader
   f) Other, please specify ____

1b. [IF YES ABOVE to option b, ASK] Who tried to change your gender identity? (check all that apply)

   a) Your choice
   b) Parent
   c) Caregiver
   d) Counselor
   e) Community leader
   f) Other, please specify ____

2. * In some cases, attempts to change sexual orientation or gender identity includes more organized activities that are sometimes referred to as "conversion therapy". Have you ever been exposed to any of the following conversion efforts? (check all that apply)

- Conversion efforts by a licensed health professional (psychologist, psychiatrist, doctor)
- Conversion efforts by an unlicensed counselor (life coach, family coach, success coach)
- Conversion efforts in a camp
- Conversion efforts by a faith-based organization
- Conversion efforts by an individual religious leader (i.e., not through a formal organization)
- Conversion efforts by another religious individual
- Other conversion efforts; please describe:

3. At what age did you first experience conversion efforts? ________

4. For how long did you experience conversion efforts?

© 2025 Gibb JK et al. *JAMA Network Open*.

- Less than 1 month
- 1 month to 1 year
- More than 1 year

5. How many times did you experience conversion efforts?
   - 1 time only
   - 2 times
   - 3 times
   - 4 times
   - 5+ times
   - Prefer not to answer.

6. At what age did you last experience conversion efforts? _______

7. Do you have anything you would like to share regarding your experiences with conversion efforts? [open respondent supplied response]

© 2025 Gibb JK et al. *JAMA Network Open*.
