# Supplementary material for: Conversion Therapy Exposure and Elevated Cardiovascular Disease Risk
Source: JAMA Netw Open. 2025 May 6;8(5):e258745. doi: 10.1001/jamanetworkopen.2025.8745 (PMC12056572; doi:10.1001/jamanetworkopen.2025.8745)
Supplement: Supplement 2. — Data Sharing Statement [file jamanetwopen-e258745-s002.pdf]

Data Sharing Statement

Gibb. Conversion Therapy Exposure and Elevated Cardiovascular Disease Risk. *JAMA Netw Open*. Published May 06, 2025. doi:10.1001/jamanetworkopen.2025.8745

Data
Data available: Yes
Data types: Deidentified participant data
How to access data: https://www.icpsr.umich.edu/web/NAHDAP/studies/37603
When available: With publication

Supporting Documents
Document types: None

Additional Information
Who can access the data: during annual updates
Types of analyses: for research purposes
Mechanisms of data availability: without support
